# Natural and Modified Oligonucleotide Sequences Show Distinct Strand Displacement Kinetics and These Are Affected Further by Molecular Crowders

**DOI:** 10.3390/biom12091249

**Published:** 2022-09-06

**Authors:** Ivana Domljanovic, Alessandro Ianiro, Curzio Rüegg, Michael Mayer, Maria Taskova

**Affiliations:** 1 Laboratory of Experimental and Translational Oncology, Department of Oncology, Microbiology and Immunology, Faculty of Science and Medicine, University of Fribourg, Chemin du Musée 18, PER17, 1700 Fribourg, Switzerland; 2 BioPhysics, Adolphe Merkle Institute, University of Fribourg, Chemin des Verdiers 4, PER 18, 1700 Fribourg, Switzerland

**Keywords:** nucleic acids, strand displacement, hybridization, FRET

## Abstract

DNA and RNA strand exchange is a process of fundamental importance in biology. Herein, we used a FRET-based assay to investigate, for the first time, the stand exchange kinetics of natural DNA, natural RNA, and locked nucleic acid (LNA)-modified DNA sequences in vitro in PBS in the absence or presence of molecular additives and macromolecular crowders such as diethylene glycol dimethyl ether (deg), polyethylene glycol (peg), and polyvinylpyrrolidone (pvp). The results show that the kinetics of strand exchange mediated by DNA, RNA, and LNA-DNA oligonucleotide sequences are different. Different molecular crowders further affect the strand displacement kinetics, highlighting the complexity of the process of nucleic acid strand exchange as it occurs in vivo. In a peg-containing buffer, the rate constant of displacement was slightly increased for the DNA displacement strand, while it was slightly decreased for the RNA and the LNA-DNA strands compared with displacement in pure PBS. When we used a deg-containing buffer, the rate constants of displacement for all three sequences were drastically increased compared with displacement in PBS. Overall, we show that interactions of the additives with the duplex strands have a significant effect on the strand displacement kinetics and this effect can exceed the one exerted by the chemical nature of the displacement strand itself.

## 1. Introduction

Homologous DNA recombination includes DNA strand exchange and occurs in all forms of life in the context of DNA repair and genetic stability [1]. Various reports show that in living organisms, recombination enzymes, such as RecA in prokaryotes or its homolog Rad51 in eukaryotes, catalyze the process of strand displacement [2,3]. Although the details of this enzymatically mediated strand displacement process of duplex DNA are only partially understood, it is proposed that the DNA strand exchange involves at least three sequential steps. First, a single strand (ss) DNA diffuses in proximity of the double-strand (ds) DNA; second, the displacement strand interacts with one strand of the double-strand DNA and leads to an exchange of base pairing of a few bases; third, the strand is exchanged along the DNA duplex [2,3].

DNA strand displacement is becoming an essential process in nanotechnology applications as well. DNA is recognized as material for nanoscale engineering due to its specificity and programmability controlled by Watson-Crick canonical base pairing [4]. These features allow the use of DNA for the construction of extremely precise nanostructures through the programmed hybridization of complementary strands [5,6]. Initially, DNA-based nanotechnology focused on the self-assembly of static structures. Subsequently, the field blossomed with the development of the DNA origami method, empowering the design and generation of “dynamic” structures. The function of these structures can be controlled and modulated by conformational changes induced by external stimuli or strand-displacement reactions [7]. For instance, dynamic DNA structures, which are based on a toehold-mediated strand displacement, [8] are used in different applications, including artificial molecular machines and programmable scaffolds [9]. In particular, the approach of toehold-mediated strand displacement is based on the attachment of an invader strand to the toehold present on the duplex, followed by stepwise dislocation of one strand of the substrate duplex until full hybridization of the complementary invader to the substrate strand is completed [10,11].

There is a need for a better understanding of the strand displacement process to reveal further fundamental mechanisms of these interactions, which are potentially important in vivo. In addition, controlled manipulation of the strand displacement process in vitro can increase the rate of the displacement reaction and the yield of assembled nanostructures, which will accelerate further applications in nanotechnology. Feng et al. reported increased DNA strand exchange rates in concentrated aqueous solutions of polyethylene glycol (peg) [12]. In the following study, the same authors highlighted that base stacking destabilization and nucleation-promoted DNA strand invasion are two core mechanisms that contribute to the catalytic role of the two semi-hydrophobic crowders used, peg and 1,2-dimethoxyethane (dme) [13]. Very recently, Norden et al. used a mismatched duplex on different positions and showed that the mismatch is displaced faster into a matched duplex with a matched DNA strand if a peg is present as a crowder [14]. They concluded that the hydrophobic interactions between the peg and the oligonucleotides are likely the cause of this catalytic effect and excluded molecular crowding as a possible mechanism.

**Figure 1:**
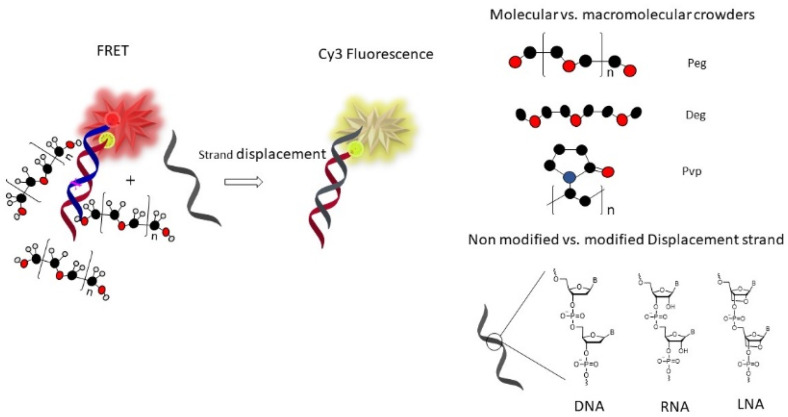
The key principle of the Föster resonance energy transfer (FRET) assay used in this study to investigate strand exchange. Initially, the duplex (ds) is labeled with a FRET pair of the fluorescent dyes Cyanine 3 (Cy3) and Cyanine 5 (Cy5). FRET occurs because of the close contact between the donor (Cy3) and the acceptor (Cy5) dyes. In particular, energy is transferred from the Cy3 to the Cy5 fluorophore through dipole-dipole interactions. As a result, the fluorescence emission from Cy5 is maximal. The addition of various (DNA, RNA, DNA-LNA) single displacement strands (DS) in different buffer conditions leads to the formation of a new duplex causing dissociation of the original duplex, resulting in a decreased FRET and decreased Cy5 fluorescence emission. These events were monitored over time to quantify the kinetics of strand exchange. Peg is polyethylene glycol, deg is diethylene glycol dimethyl ether, pvp is polyvinylpyrrolidone, and LNA is locked nucleic acid.

Herein, we investigated and compared, for the first time, the strand exchange kinetics and the base-pairing fidelity of natural DNA, natural RNA, and LNA-modified DNA sequences as displacement strands. We included diethylene glycol dimethyl ether (deg), which could help solubilize DNA via hydrophobic interactions without exerting a crowding effect due to the small size, and the macromolecular crowders peg and polyvinylpyrrolidone (pvp) (Figure 1) to model crowding inside living cells.

Our results indicate that the different chemical natures of the strands, including DNA, RNA, or LNA-DNA, have distinct displacement kinetics in pure PBS, with the LNA-DNA showing the fastest displacement. The different buffer additives used further affected the kinetics to variable degrees. Overall, we show that concerning the strand displacement kinetics, interactions of the additives with the hybridized strands have a big effect that can exceed the effect exerted by the nature of the displacement strand itself.

## 2. Materials and Methods

Reagents and solvents were obtained from commercial suppliers and used without further purification. The oligonucleotide sequences were supplied by Biomers (Ulm, Germany) and used as received. Polyethylene glycol (peg_6000), diethylene glycol dimethyl ether (deg), and polyvinylpyrrolidone (pvp_360,000) were purchased from Sigma (Buchs, Switzerland). We annealed the final master mix using an oligonucleotide concentration of 1 µM for each strand in the total volume of 100 µL.

### 2.1. Sample Preparation and Fluorescence Measurements

Annealing of the FRET pair duplex oligonucleotides (Bmut-CoBmut, 1000 nM in a 100 µL) was performed by exposing the mixture to 90 °C for 10 min and cooling down at room temperature for 2 h, followed by chilling at +4 °C for 1 h. Once the duplex Bmut-CoBmut was formed, it exhibited FRET upon excitation. Next, the crowders, peg 45%, deg 45%, and pvp 10% (concentrations given as *w*/*w*) were introduced for a minimum time of 15 min. Immediately after the addition of the displacement strand (DS1, DS2, DS3, or DSCtrl, in a five-fold molar excess), the Cy5 fluorescence emission over time was measured on a BioTek Cytation 5 Imaging Multi-Mode reader for a minimum of 30 min. To monitor FRET, we excited the donor Cy3 dye at 535 nm and followed the fluorescence emission of the acceptor Cy5 at 675 nm. We measured the kinetics in a 37 °C temperature-controlled instrument. As the displacement reaction proceeds, the FRET pair dissociates, resulting in a decrease in the FRET efficiency between Cy3 and Cy5, and decreased Cy5 fluorescence emission.

### 2.2. Circular Dichroism (CD) Measurements

CD measurements were performed on a Chirascan CD spectropolarimeter. The ds (2 µM from each strand) was annealed as described above, and the crowders were introduced for a minimum time of 15 min before the measurements. The CD measurements were performed at 37 °C using bandwidth of 1 nm and time per point of 1 s.

### 2.3. Viscosity Measurements

Viscosity measurements were performed with a 50 mm diameter cone and plate geometry mounted on a stress-controlled rheometer (MCR300, Paar Physica, Anton Paar, Virginia, NB, USA). The sample temperature of 37 °C was maintained constant by a Peltier module. For each solution, the value of the viscosity was obtained from the ratio between the measured stress and the imposed shear rate for shear rates in the range of 10–1000 1/s. As the viscosity is independent on the shear rate for each solution, we reported the mean and standard deviations for different shear rates. The kinetic constants determined from the data fitting were multiplied by the buffer viscosity (Table S1) to calculate the viscosity-independent constants reported in Figure S4. In fact, it is known from collision theory that the kinetic constant of diffusion-governed bimolecular processes depends on the inverse of solvent viscosity [15].

### 2.4. Kinetic Modelling and Curve Fitting

A simple kinetic model describing an irreversible displacement reaction was used to analyze the experimental data:

(1) $${\mathrm{AS}}_{1}+{\;S}_{2}\overset{k}{\to }{\mathrm{AS}}_{2}+{\;S}_{1}$$

In Equation (1), ${\mathrm{AS}}_{1}$ indicates the original duplex, while $S_{2}$ is the displacement strand and k is the displacement constant with units L·mol^−1^·min^−1^. This reaction scheme can be described by the following rate equation:

(2) $$\frac{d\left[{\mathrm{AS}}_{1}\right]}{dt}=\frac{d\left[S_{2}\right]}{dt}=-\frac{d\left[{\mathrm{AS}}_{2}\right]}{dt}=-\frac{d\left[S_{1}\right]}{dt}=-k\left[{\mathrm{AS}}_{1}\right]\left[S_{2}\right],$$

where the brackets indicate concentrations and t is time. If k is known, Equation (2) can be numerically integrated under the constraints that ${\left[{\mathrm{AS}}_{1}\right]}_{t=0}=1$ µM, ${\left[S_{2}\right]}_{t=0}=5$ µM, $\;{\left[{\mathrm{AS}}_{2}\right]}_{t=0}=0$, $\;{\left[S_{1}\right]}_{t=0}=0$, corresponding to our initial experimental conditions. However, k is unknown. To determine k, we, therefore, embedded the numerical integration routine into a fitting algorithm. The fitting algorithm uses an initial guess for the value of k to numerically determine the value of $\left[{\mathrm{AS}}_{1}\right]$ as a function of time. The obtained $\left[{\mathrm{AS}}_{1}\right]\;$ curve is then normalized with respect to ${\left[{\mathrm{AS}}_{1}\right]}_{t=0}$ and compared to the normalized fluorescence intensity $I/I_{t=0}$ under the assumption that the measured fluorescence intensity is proportional to $\left[{\mathrm{AS}}_{1}\right]$. The procedure is repeated iteratively by varying  k until the k value that minimizes the difference between numerically predicted and experimental data is found.

The fitting algorithm was based on Python’s LMFIT (https://lmfit.github.io/lmfit-py/, 3 September 2022) package using a nonlinear least squares method (‘leastsq’ method in scipy). The numerical integration of the displacement rate equation was performed using the scipy.integrate solve_ivp module (https://docs.scipy.org/doc/scipy/reference/generated/scipy.integrate.solve_ivp.html, 3 September 2022) with the explicit Runge-Kutta method of order 8 (‘DOP853’). The same procedure has been used to test more comprehensive kinetic models comprising multiple reversible steps. However, increasing the number of fitting parameters quickly resulted in overfitting, fitting instabilities, and a strong dependence of the fitting results from the initial guesses. Hence, only the results obtained with the single-parameter model (Equations (1) and (2)) were deemed reliable and are reported in this work.

## 3. Results

### 3.1. Design of Sequences and FRET Assay

For our study, we chose the *BRAF V600E* mutated gene as a model design for the duplex DNA (ds). The V600E mutated BRAF kinase acts as driver oncogene in many cancers, particularly melanoma and colorectal cancer, and can be therapeutically targeted using small kinase inhibitors [16].

We selected a part of the gene (Bmut) with a length of 20 nucleotides that contains the single point mutation on the 16th position and designed it to contain Cyanine 5 (Cy5) fluorescent dye on the 5′ terminus. Next, we designed a complementary 20^mer^ sequence (CoBmut) to the Bmut labeled with Cyanine 3 (Cy3) fluorescent dye on the 3′ terminus to contain a mismatch opposite of position 16th. We included various displacement strands (DS1, DS2, and DS3) to be fully complementary to the Bmut sequence. We designed the displacement oligonucleotide sequences (DS) to be natural DNA (DS1), natural RNA (DS2), and locked nucleic acid (LNA) modified DNA sequence (DS3) (Table 1). LNA is a bicyclic modified nucleotide that is often incorporated into synthetic probes. It improves their target binding affinity and specificity [17]. For control of the displacement capacity of the system, we designed and used a non-matching control displacement sequence (DSCtrl).

The fluorescent dyes Cy3 and Cy5, when covalently attached to the end of a duplex DNA, show a planar capping configuration similar to that of an additional base pair [18]. This configuration imparts stability to the duplex and increases the fluorescence quantum yield and lifetime [18]. Furthermore, the hybridization of two complementary strands labeled with dyes with overlapping emission and absorbance properties results in Förster resonance energy transfer (FRET) [19]. Cy3 and Cy5 dyes work as FRET donor and acceptor pairs, respectively, and we used FRET to monitor the strand exchange process. To quantify the kinetics of displacement, we calculated the strand exchange rate constant k, (L·min^−1^·mol^−1^) by fitting the experimentally measured and normalized kinetic traces of fluorescence intensity. The fitting model is based on a simple, irreversible displacement reaction (1). Although this model provides a simplified picture of the displacement process, it is preferred over more detailed multi-parameter kinetic models to avoid overfitting. Details of the fitting procedure can be found in the Materials and Methods Section 2.4.

### 3.2. Strand Displacement Kinetics

**Figure 2:**
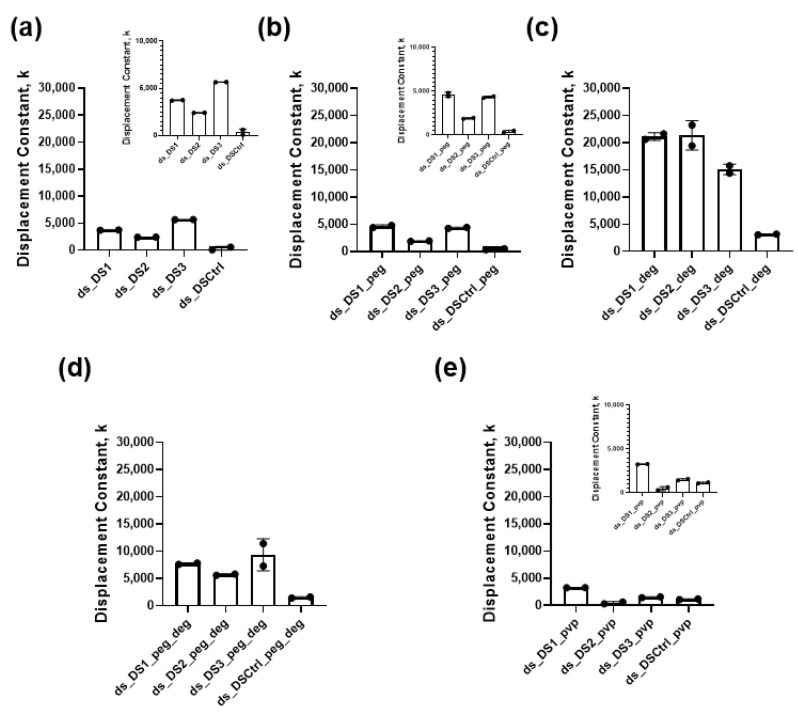
Rate constants of strand displacement k, (L·min^−1^·mol^−1^) for the duplex (Bmut-CoBmut) displaced with DS1, DS2, DS3, and DSCtrl in (**a**) pure PBS buffer or PBS buffers enriched with (**b**) polyethylene glycol (peg), (**c**) diethylene glycol dimethyl ether (deg), (**d**) mixed peg-deg or (**e**) polyvinylpyrrolidone (pvp) as indicated. The displacement strands (DS1. DS2, DS3, and DSCtrl) are represented in Table 1. Error bars indicate ± SD (*n* = 2). For assessment of the data between the different displacement strands in one particular buffer condition, we used one-way ANOVA and calculated *p* < 0.05. For comparison of the data between the displacement strands in two different buffers, we used two-factor ANOVA with replication and calculated *p* < 0.05.

From the time course of the monitored fluorescence, we observed a decrease in the Cy5 fluorescence emission intensity because of strand displacement and separation of the Cy3-Cy5 FRET pair (Figure S1). In pure phosphate-buffered saline (PBS), the highest rate of decay of the Cy5 fluorescence emission intensity was observed for the LNA-DNA strand (DS3), followed by the natural DNA (DS1) and then by the natural RNA (DS2) strand (Figure S1). In particular, the strand displacement constant for DS3 is 5670 L·min^−1^·mol^−1^ and for DS1 is 3744 L·min^−1^·mol^−1^ in pure PBS (Figure 2a, *p* < 0.05). The DS2 shows the lowest constant for strand displacement of 2420 L·min^−1^·mol^−1^ in pure PBS, as indicated by the smallest reduction in Cy5 fluorescence emission intensity at the end time of the measurements. As expected, the experiments with the control sequence displacement strand (DSCtrl) show statistically significant low displacement constants in pure PBS. Using a higher salt concentration buffer (addition of 150 mM NaCl), all three displacement strands showed slightly decreased displacement kinetics, but this reduction was statistically insignificant (Figure S3a, *p >* 0.05). Nonetheless, the position of the individual rate constants in PBS buffer that contains an additional 150 mM NaCl was similar to in PBS buffer. In particular, the DS3 showed the fastest and the DS2 the slowest displacement kinetics (Figures S2 and S3a).

When introducing macromolecular peg as a crowder in the buffer system, DS1 and DS3 showed similar rates of decay of the Cy5 fluorescence emission intensity (i.e., similar rates of strand displacement) that were higher compared with the rates of decay of the DS2. Noteworthy, in the buffer that contains peg crowder, while the displacement constant of DS1 was increased by approximately 23% compared with pure PBS, the displacement constants of DS2 and DS3 were decreased by approximately 21% and 23%, respectively, compared with pure PBS (Figure 2a,b). In addition, the effects of increased salt concentration (addition of further 150 mM NaCl) in the buffer that contains peg crowder were investigated (Figures S2 and S3). Similar to pure PBS, the slight reduction of the rate constants of displacement in higher-salt peg-buffer compared with peg-buffer was not statistically significant (*p >* 0.05).

When using the buffer containing deg, the strand displacement kinetics increased strongly for all three-displacement strands. In this case, the DS1 and DS2 showed similar displacement kinetics that were faster than the displacement kinetics of DS3 (Figure 2c). Following the displacement constants, DS1 in deg-buffer exchanged 5.6-fold faster compared to pure PBS and 4.6-fold faster compared to peg-buffer. The strand exchange kinetics of DS2 in deg-buffer was even more distinct, with an 8.8-fold faster exchange compared to pure PBS and an 11.1-fold faster exchange compared with peg-buffer. In the case of DS3, this effect was smaller with a 2.8-fold faster exchange in deg-buffer compared with pure PBS and a 3.6-fold faster exchange compared with peg-buffer (Figure 2). In addition, the slightly slower displacement of the DS1 and DS2 and the slightly faster displacement of the DS3 in a higher-salt concentration deg-buffer compared with deg containing buffer was statistically non-significant (*p >* 0.05).

Since the cellular environment is a complex mixture of molecules and macromolecules, we performed the experiments in a buffer containing both peg and deg. Using this condition, DS3 showed the highest rate of the fluorescence decay, followed by DS1 with intermediate and DS2 with the slowest rate of decay (Figure S1). Following the displacement constants, while the DS1 in the buffer containing peg-deg mixture showed a 2.7-fold decrease in the displacement constant compared to the deg-buffer, still the displacement constant in the peg–deg buffer was 2.1 times higher compared with PBS and 1.7 times higher compared with the peg-buffer. Nevertheless, the DS2 displayed a 3.7-fold decrease in the displacement constant in the peg-deg buffer when compared with the deg-buffer. The rate constant of DS2 in the mix buffer was 2.4- and 3.0-fold higher than in the PBS and the peg-buffer, respectively. DS3 showed a 1.7-fold decrease in the displacement constant in the mix buffer compared with the deg-buffer. Still, the displacement constant of DS3 in the peg-deg buffer was 1.7 times higher compared with the PBS and 2.2 times higher compared with the peg-buffer. When looking at the results for higher salt concentration buffers, DS1 and DS3 again showed faster displacement compared with DS2. However, the difference in the displacement constants for all three displacement strands in peg-deg buffer with and without additional salt was statistically non-significant (*p >* 0.05).

We completed experiments in pvp-buffer, using pvp as a structurally different macromolecule than peg. In general, we observed decreased kinetics for strand displacement in the pvp-buffer compared with pure PBS. All three displacement strands showed very slow rates of decay in pvp-buffer, with the DS2 showing the lowest displacement constant and the DS1 the highest (Figure 2e). The displacement constants in higher salt pvp-buffer did not show a statistically significant (*p >* 0.05) difference with the displacement constants in pvp-buffer (Figure S3e).

In addition, to take into account the viscosity of the different crowder buffers, we normalized the displacement constants with the measured viscosity (Table S1). Overall, Figure S4 shows that in this case, all buffers have increased displacement constant compared with pure PBS. Moreover, when we consider the viscosity, the highest rate of displacement was observed in the peg-deg buffer.

**Figure 3:**
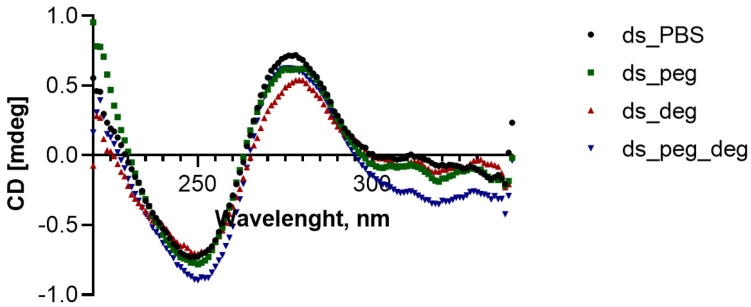
Circular dichroism (CD) spectra of the ds (Bmut-CoBmut) without fluorescent dyes in four different buffer conditions (pure PBS, peg buffer, deg buffer, and mixed peg_deg buffer). Peg is polyethylene glycol, and deg is diethylene glycol dimethyl ether.

To examine the ds (Bmut-CoBmut) duplex geometry, we performed circular dichroism (CD) studies in various buffer conditions. For these studies, we used the same duplex sequences without labeled fluorescent dyes Cy3 and Cy5. The CD spectra confirmed that there is no significant distortion of the duplex geometry in all buffer conditions (Figure 3). Notably, the CD spectrum in the pure PBS is similar to the other conditions with crowder-enriched buffers. The spectrum shows a positive peak at 280 nm and a negative peak at 250 nm, which is expected for a B-DNA conformation of a double-stranded DNA [20].

## 4. Discussion

In this work, we studied the strand exchange kinetics of three different oligonucleotides: DNA (DS1), RNA (DS2), and LNA modified DNA sequence (DS3) in various buffers with or without molecular and macromolecular crowders of different sizes to simulate a semi-hydrophobic (peg and deg) environment in vitro. In addition to strand exchange phenomena between double-stranded and single-stranded DNA occurring naturally during homologous DNA recombination, the CRISPR-Cas-based gene editing machinery also involves RNA-DNA recognition and duplex formation [21]. Moreover, many LNA-containing DNA sequences are employed in drug discovery studies, such as oligonucleotide therapeutics [22] and advanced modified sgRNA for CRISPR-Cas [23].

Our data indicate that the kinetic strand displacement depends on the nature of the newly introduced strand and on the nature of the crowders. In general, comparing the three displacement strands, the displacement capacity of DS3 was the least affected by the different buffer conditions. More specifically, in a pure PBS, the LNA-DNA oligonucleotide (DS3) can convert the mismatched duplex into a matched one fastest and to a greater extent compared with the natural DNA (DS1) and RNA (DS2) sequence. These results suggest that when a DNA strand contains LNA modifications, its base-pairing fidelity and strand displacement kinetics increase. These findings are consistent with previously reported properties of the LNA modification introduced to a DNA sequence to increase the target binding specificity [24,25,26].

However, in the peg-containing buffer, DS1 and DS3 showed similar and faster displacement kinetics compared to DS2. In this buffer condition, while the displacement kinetics of DS1 was slightly increased, it was slightly decreased for DS2 and DS3 when compared with the PBS condition. Nordèn et al. previously reported that a matched DNA strand could displace a mismatch strand in a duplex faster in a solution containing non-ionic semi-hydrophobic peg molecules [14]. The authors excluded that the cause for this effect is peg-induced variations in the DNA duplex conformation or duplex melting. Instead, they proposed a hydrophobic effect mechanism that includes three different steps. First, interruption of the base stacking and strand invasion; second, stronger hydrogen bonds by decreased water activity; and third, stabilization of the mismatch by intercalation of the hydrophobic crowder into the mismatched gaps [14]. In agreement with this report, we confirm slightly faster displacement kinetics of DNA displacement strands in a peg-buffer. Nevertheless, the additional data on DS2 and DS3 displacement strands reveal that peg-enriched buffer fails to increase the displacement kinetics of RNA and LNA-DNA displacement strands.

Notably, the data from the experiments performed in a buffer containing deg reveal a strong increase in the rate constants for all three displacement sequences. Specifically, this increase was more pronounced for the DS2 and the DS1 and somewhat smaller for the DS3. Structurally, deg is related to peg and provides a similar semi-hydrophobic environment without volume exclusion (i.e., no crowding effect) [13]. For its structure and size, deg can be regarded as a co-solvent rather than a crowder, whose interactions with the nucleobases within the duplex (Bmut-CoBmut) facilitate the mismatched duplex displacement [27]. These results confirm that hydrophobic interactions dominate crowding in enhancing displacement kinetics. The slight increase in the rate constant of displacement of the control sequence (DSCtrl) in deg buffer (Figure 2c) shows that deg is interacting relatively strongly with the original duplex (ds), possibly changing the dipole-dipole interactions between the dyes and hence the FRET effect.

Since the different displacement strands showed distinct displacement kinetics in the different buffers and considering that the intracellular environment is a complex mixture of small molecules and macromolecules, [28,29] we measured the rate of the displacement kinetics in a mixed peg-deg buffer. Interestingly, for all three oligonucleotides, the rate of decay was faster compared with the peg-buffer and slower compared with the deg-buffer. These results suggest that there is a balance between solubility and crowding effects, with solubility exerting the dominant contribution.

Using the same experimental conditions, we investigated the strand displacement ability of all three oligonucleotides in a buffer containing pvp. We chose pvp because it is a structurally different macromolecule from peg and because of its polydisperse role as a crowder [30]. Pvp is a biomaterial that is also used in tissue engineering and for the expansion of progenitor cells in vitro [30,31,32]. From the time traces of strand displacement, it is evident that the rate of Cy5 fluorescence decay in pvp-buffer does not exceed the one in pure PBS. This observation agrees with previously reported results on macropolymers such as dextran, which lack the ability to increase the strand displacement constant of a DNA displacement strand. In addition, the displacement control sequence (DSCtrl) showed insignificant displacement kinetics in all different buffer conditions confirming the sequence specificity of the displacement process.

Previous reports showed that the duplex stability and melting temperature (*T_m_*) do not directly correlate with the rate of strand displacement by DNA strand in pure and peg-buffers. Specifically, increasing the NaCl concentration in the buffer compensates for the possible lower *T_m_* of the duplex caused by the peg crowder [13]. Our data on the stabilized duplex (by addition of an extra 150 mM NaCl) did not show a change in the rank order of the strand displacement kinetics compared to the ones shown by DS1, DS2, and DS3 in the various buffers without additional salt. Moreover, the CD spectra confirm that the ds have overall B-DNA conformation in all crowder-enriched buffers, similar to pure PBS. The overall data indicate that even though the different crowders may react and intercalate within the duplex to affect the strand displacement ability, they do not directly destabilize and disturb the original duplex geometry.

As a final consideration, while we used a simplified single-step kinetic model to derive rate constants of displacement, the displacement process occurs in multiple steps, the first of which is diffusional. In conditions in which the diffusional step is rate limiting (e.g., low concentration of strands), the solvent viscosity can play an increasingly important role in the overall kinetics of the process. For this reason, we normalized the displacement kinetics by the solvent viscosity (Table S1 and Figure S4). As a result of this normalization, all buffers show increased rate constants of displacement compared to pure PBS, with the highest rate of displacement found in the peg-deg buffer. These results might suggest a cooperative effect between crowding and solubility, which is counteracted by the increase in solvent viscosity. Further investigations, a deeper mechanistic understanding of the displacement process, and a more comprehensive multi-step kinetic model will be required to verify this hypothesis.

## 5. Conclusions

Oligonucleotide sequences of different chemical nature (DNA, RNA, LNA-DNA) show distinct duplex strand displacement kinetics in pure PBS buffer. Among the three displacement sequences studied, the LNA-DNA showed the fastest displacement in PBS, followed by DNA and then by RNA. The displacement kinetics can be influenced by the introduction of different crowders to the buffer. While a peg-enriched buffer slightly increases the displacement kinetics for DNA, it decreases the kinetics of the RNA and LNA-DNA displacement strands. Importantly, the addition of deg to PBS buffer increases the displacement kinetics of all three-displacement sequences. The data herein suggest that the intracellular environment might affect the in vivo strand displacement and the base-pairing fidelity via local hydrophobic interactions and crowding. These two mechanisms are inter-related in a complex fashion, with anticipation of the hydrophobic effect being the dominant one. Finally, the results suggest that in regard to displacement kinetics, the effect of the crowders might also be dominant over the nature of the displacement strand.

## Figures and Tables

**Table 1:** Oligonucleotide sequences used in this study.

Name	Sequence
Bmut	/Cy5/TT TGG TCT AGC TAC AG**A** GAA
CoBmut	TTC **C**CT GTA GCT AGA CCA AA/Cy3/
DS1	TTC TCT GTA GCT AGA CCA AA
DS2	rUrUrC rUrCrU rGrUrA rGrCrU rArGrA rCrCrA rArA
DS3	TTC +T+CT GTA GCT AGA C+C+A AA
DSCtrl	TTG CAT CGT CAC AAA AGT GAT C

The duplex (ds) consists of Bmut-CoBmut. Cy5 fluorescent dye is attached to the 5′ terminus of the Bmut. Cy3 fluorescent dye is attached to the 3′ terminus of the CoBmut sequence. DS1, DS2, DS3, and DSCtrl are different displacement strands. Mismatched nucleotides in mutated oncogene (Bmut and CoBmut) are in bold, and oligonucleotides with LNA in the sequence are indicated with a “+” in front of the nucleotide. LNA is locked nucleic acids.

## Data Availability

Not applicable.

## Supplementary Materials

The following supporting information can be downloaded at: https://www.mdpi.com/article/10.3390/biom12091249/s1, Figure S1: Kinetic plots of Cy5 fluorescence emission in PBS and PBS containing crowders; Figure S2: Kinetic plots of Cy5 fluorescence emission in higher salt concentration PBS buffers; Figure S3: Strand exchange rate constants in higher salt concentration PBS buffers; Table S1: Measured viscosity [Pa*s] of the various PBS buffers; Figure S4: Normalized displacement constant k, (L·min^−1^·mol^−1^) with the measured viscosity; Fit results.
