# Supplementary material for: Natural and Modified Oligonucleotide Sequences Show Distinct Strand Displacement Kinetics and These Are Affected Further by Molecular Crowders
Source: Biomolecules. 2022 Sep 6;12(9):1249. doi: 10.3390/biom12091249 (PMC9496266; doi:10.3390/biom12091249)
Supplement: Supplementary file 1 [file biomolecules-12-01249-s001.zip › biomolecules-1861350-supplementary.pdf]

Supporting Information

Natural and Modified Oligonucleotide Sequences Show Distinct Strand Displacement Kinetics and These Are Affected Further by Molecular Crowders

Ivana Domljanovic[1], Alessandro Ianiro[2],Curzio Rüegg[1], Michael Mayer[2] and Maria Taskova[2]*

Contents

1. Kinetic plots of Cy5 fluorescence emission in PBS and PBS containing crowders
2. Kinetic plots of Cy5 fluorescence emission in higher salt concentration PBS buffer and PBS containing crowders
3. Strand exchange rate constants in higher salt concentration PBS buffers
4. Viscosity normalized strand exchange rate constants in PBS buffers
5. Fit results
6. Fit results in higher salt

1. Kinetic plots of Cy5 fluorescence emission in PBS and PBS containing crowders

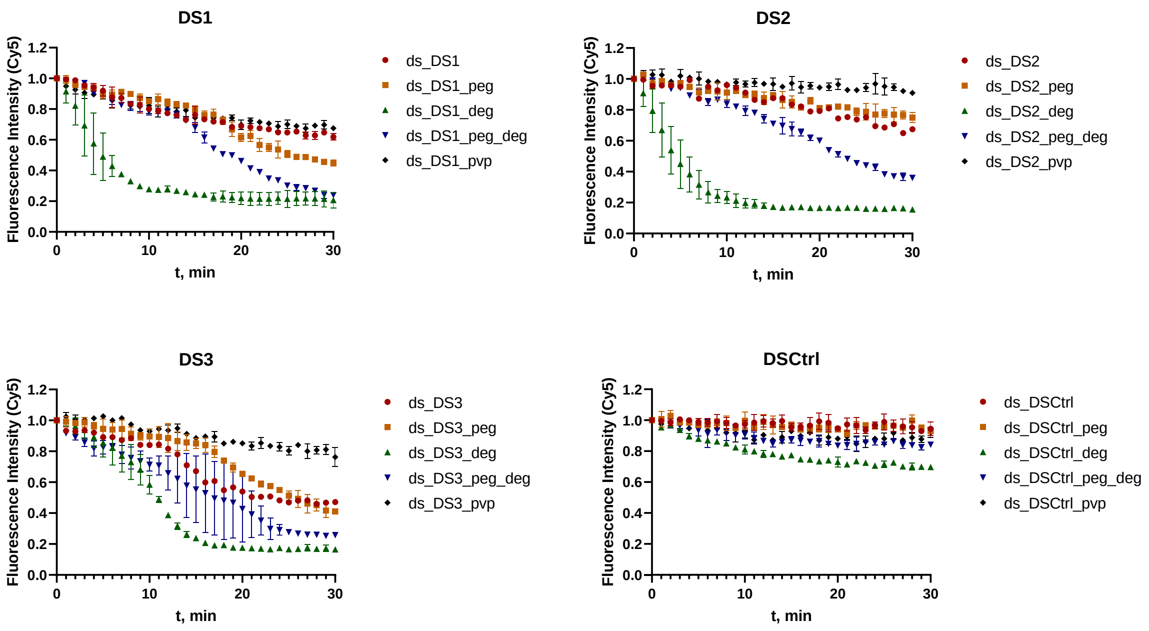

Figure S1. Fluorescence intensity Cy5 kinetic curves for duplex (Bmut-CoBmut) displaced with DS1, DS2, DS3, and DSCtrl in pure PBS buffer and buffers complexed with polyethylene glycol (peg), diethylene glycol dimethyl ether (deg), mixed peg-deg and polyvinylpyrrolidone (pvp) over time of minimum of 30 min at a temperature of 37 °C, as indicated. The displacement strands DS1, DS2, DS3, and DSCtrl are represented in Table 1. Error bars indicate ± SD (n = 2)

2. Kinetic plots of Cy5 fluorescence emission in higher salt concentration PBS buffers

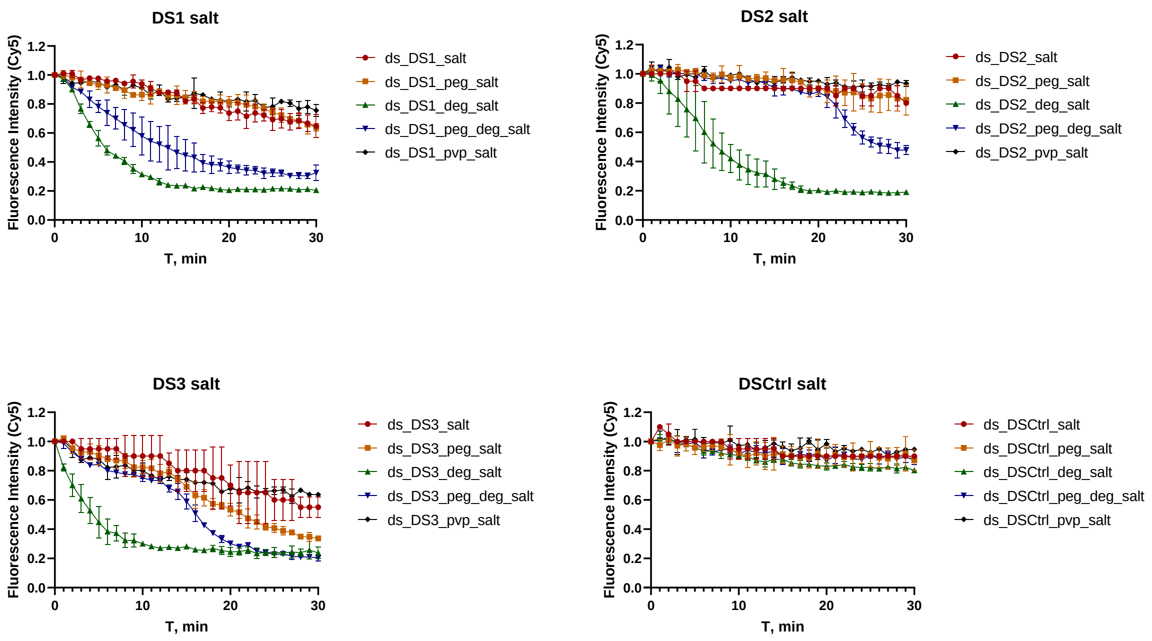

Figure S2. Fluorescence intensity Cy5 kinetic plots for duplex-ds (Bmut-CoBmut) with DS1, DS2, DS3, DSCtrl in all buffers (peg, deg, peg_deg, pvp) with higher salt concentration (additional 150 mM NaCl) as indicated. Error bars indicate ± SD (n = 2).

3. Strand exchange rate constants in higher salt concentration PBS buffers

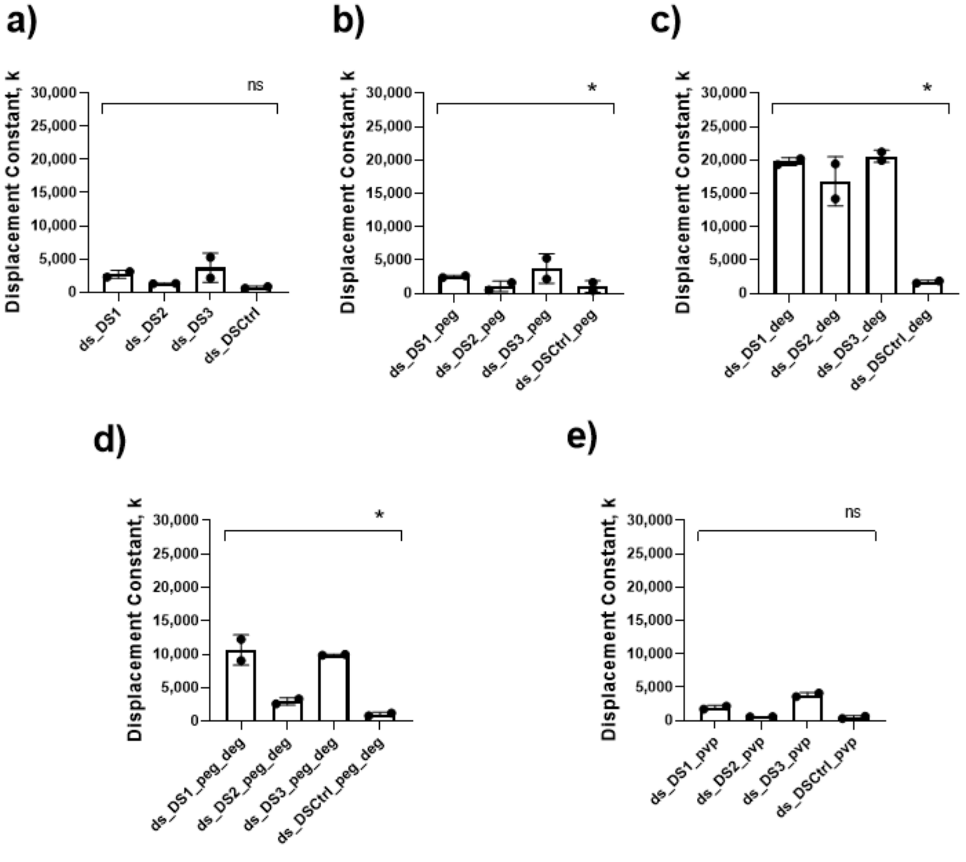

Figure S3. Displacement constant k, (L·min-1·mol-1) for duplex (Bmut-CoBmut) displaced with DS1, DS2, DS3, and DSCtrl in (a) PBS buffer with higher salt concentration (addition of 150 mM NaCl) or PBS buffers with higher salt concentration enriched with (b) polyethylene glycol (peg), (c) diethylene glycol dimethyl ether (deg), (d) mixed peg-deg and (e) polyvinylpyrrolidone (pvp) as indicated. The displacement strands DS1, DS2, DS3 and DSCtrl are represented in Table 1. Error bars indicate ± SD (n = 2). P values were calculated using one-way ANOVA. Abbreviations: "" indicates 95% confidence; "ns" indicates statistically not significant.*

4. Viscosity normalized strand exchange rate constants in PBS buffers

Table S1. Measured viscosity [Pa*s] of the various PBS buffers

	pbs	peg	deg	peg_deg	pvp
Unit	[Pa*s]				
Measurements	0.000806<br>0.000839<br>0.000693<br>0.000660<br>0.000678<br>0.001032<br>0.000809<br>0.000855<br>0.000630<br>0.000779	0.005668<br>0.005635<br>0.005698<br>0.005704<br>0.005658<br>0.005833<br>0.005753<br>0.005908<br>0.005213<br>0.006015	0.000932<br>0.000999<br>0.000911<br>0.000901<br>0.000882<br>0.001307<br>0.001109<br>0.001008<br>0.001011<br>0.00105	0.004836<br>0.004710<br>0.004782<br>0.004782<br>0.004832<br>0.005242<br>0.004835<br>0.004978<br>0.004930<br>0.004696	0.004251<br>0.004113<br>0.004264<br>0.004242<br>0.004328<br>0.004751<br>0.004386<br>0.004387<br>0.004414<br>0.005117
Average	0.000778	0.005709	0.001011	0.004862	0.004425

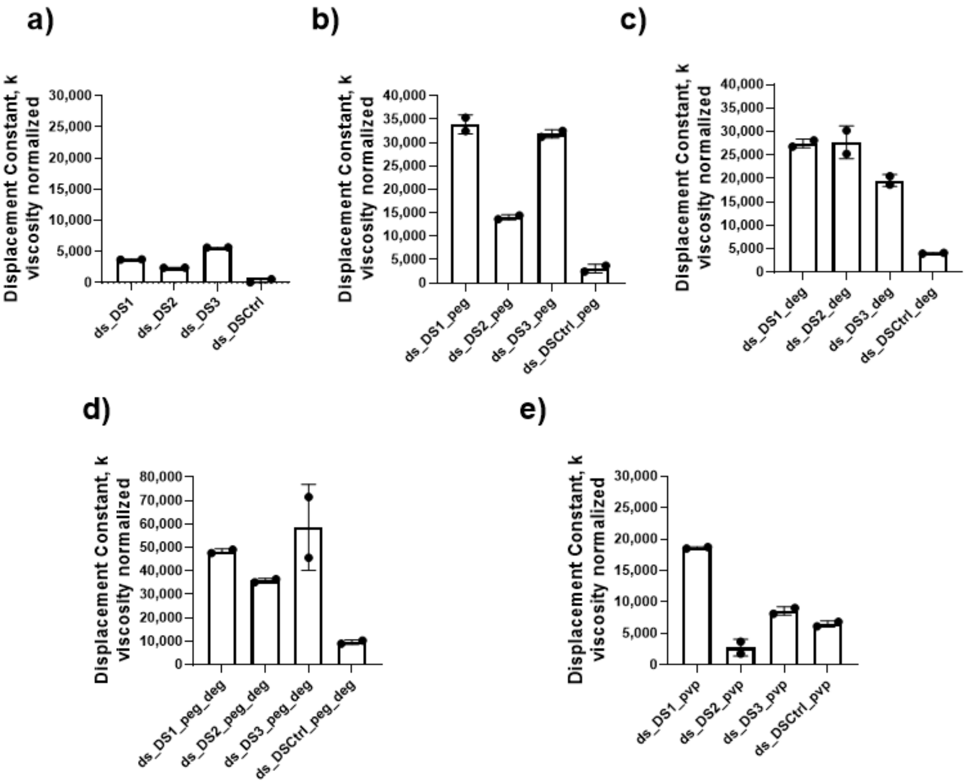

Figure S4. Normalized displacement constant k, (L·min-1·mol-1) with the measured viscosity (Table S1) for duplex (Bmut-CoBmut) displaced with DS1, DS2, DS3, and DSCtrl in (a) PBS buffer or PBS buffers enriched with (b) polyethylene glycol (peg), (c) diethylene glycol dimethyl ether (deg), (d) mixed peg-deg and (e) polyvinylpyrrolidone (pvp) as indicated. The displacement strands DS1, DS2, DS3 and DSCtrl are represented in Table 1. Error bars indicate ± SD (n = 2).

5. Fit results

Pure PBS buffer

ds_DSCtrl

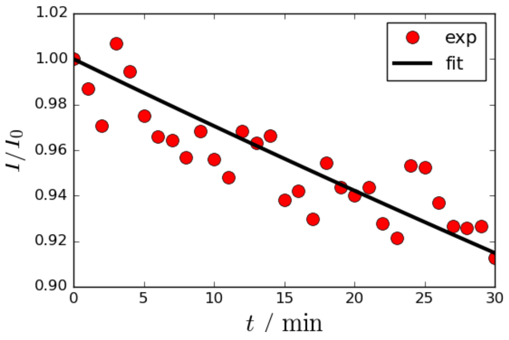

598	+/- 29 (4.9%)
95	+/- 46 (49.2%)

ds_DS1

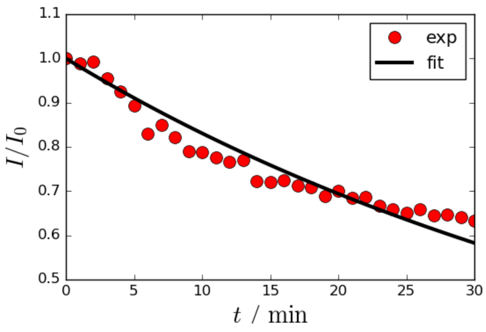

3773	+/- 105 (2.8%)
3715	+/- 75 (2.0%)

ds_DS2

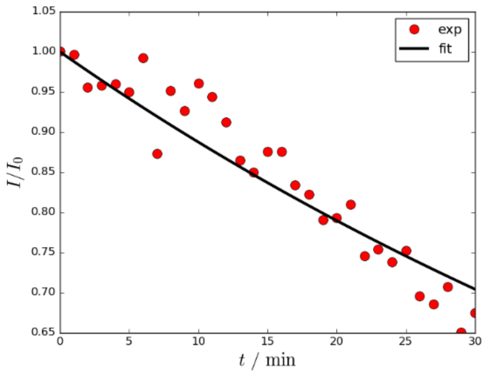

2429	+/- 96 (4.0%)
2412	+/- 97 (4.0%)

ds_DS3

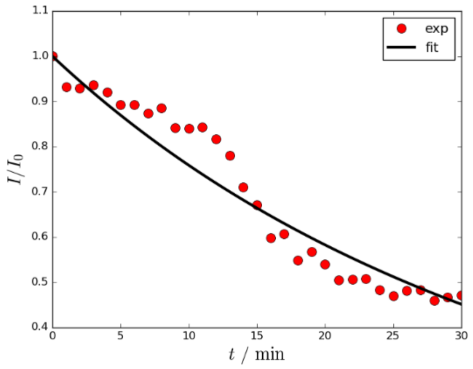

5660	+/- 202 (3.6%)
5679	+/- 202 (3.5%)

Peg buffer

ds_DSCtrl_peg

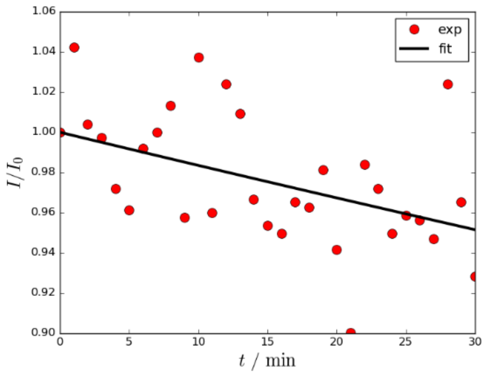

508	+/- 56 (11.1%)
332	+/- 60 (18.2%)

ds_DS1_peg

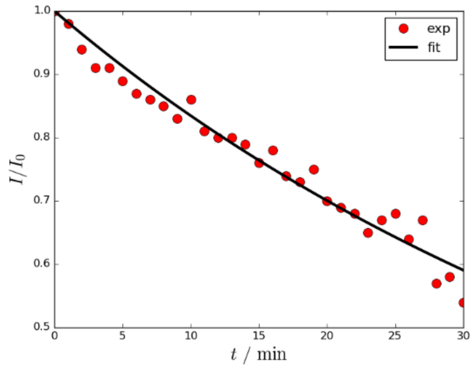

4818.501	+/- 213 (4.4%)
4424.28536	+/- 224 (5.1%)

ds_DS2_peg

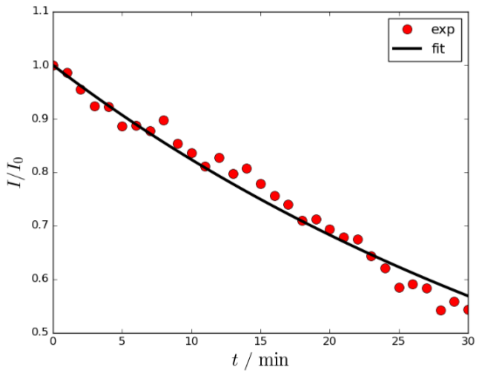

1860	+/- 66 (3.6%)
1969	+/- 70 (3.6%)

ds_DS3_peg

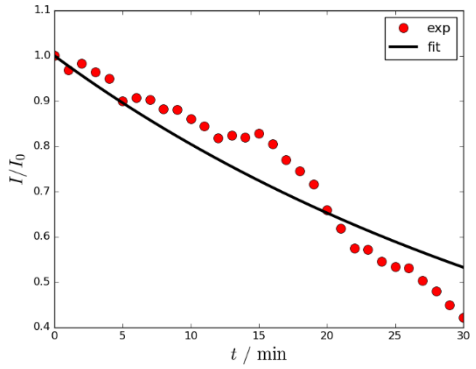

4430	+/- 211 (4.8%)
4254	+/- 380. (8.9%)

Deg buffer

ds_DSCtrl_deg

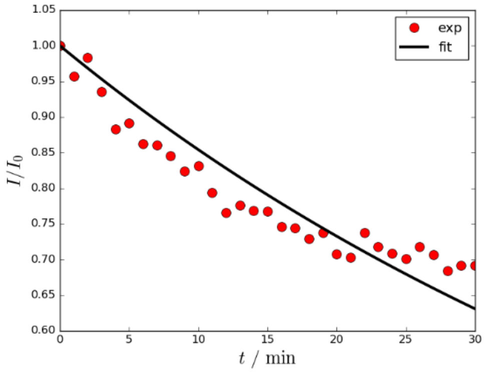

3066	+/- 114 (3.7%)
3198	+/- 110 (3.5%)

ds_DS1_deg

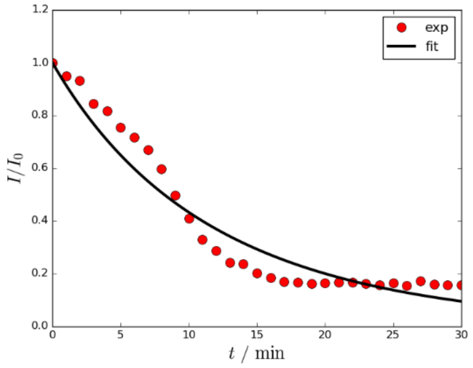

20616	+/- 1029 (5.0%)
21628	+/- 1857 (8.6%)

ds_DS2_deg

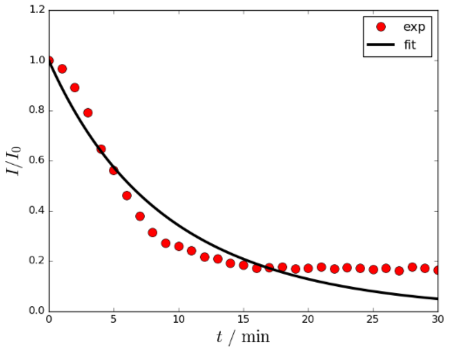

19427	+/- 1210 (6.2%)
23241	+/- 1191 (5.1%)

ds_DS3_deg

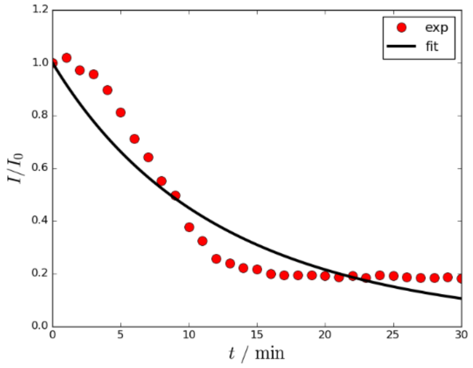

15764	+/- 720 (4.6%)
14385	+/- 1138 (7.9%)

Peg_deg buffer

ds_DSCtrl_peg_deg

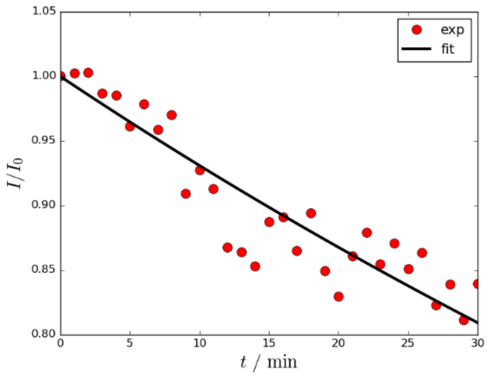

1439	+/- 58 (4.1%)
1644	+/- 103 (6.3%)

ds_DS1_peg_deg

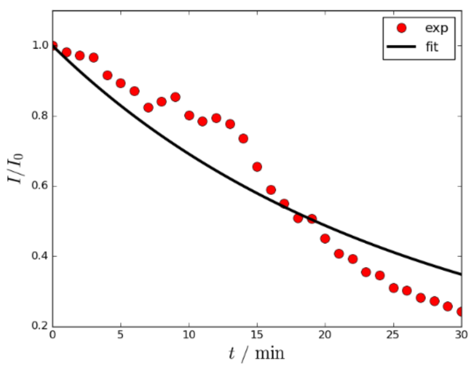

7622.631	+/- 434 (5.7%)
7857.06592	+/- 430 (5.5%)

ds_DS2_peg_deg

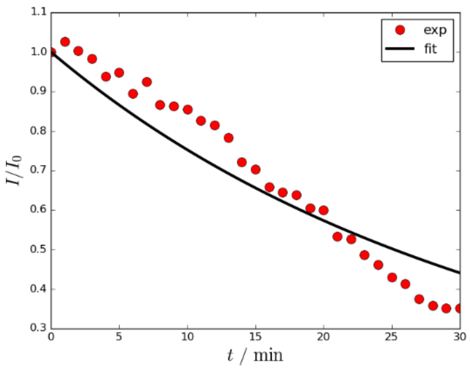

5837	+/- 290 (5.0%)
5620	+/- 260 (4.6%)

ds_DS3_peg_deg

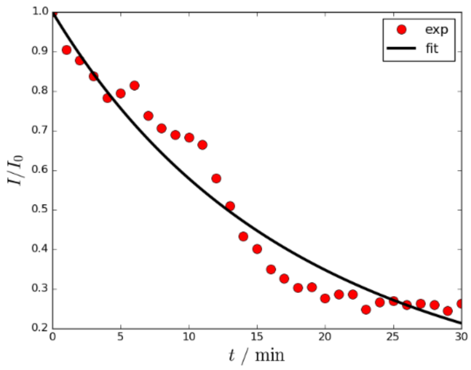

11447	+/- 391 (3.4%)
7304	+/- 438 (6.0%)

Pvp buffer

ds_DSCtrl_pvp

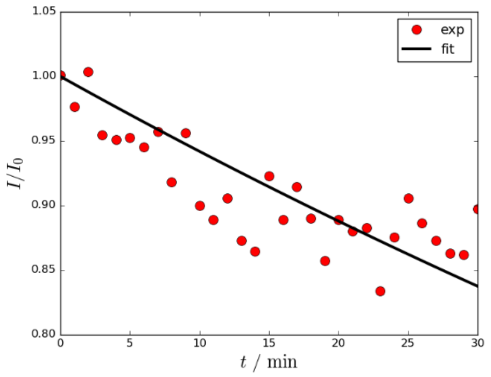

1076	+/- 102 (9.5%)
1202	+/- 70 (5.8%)

ds_DS1_pvp

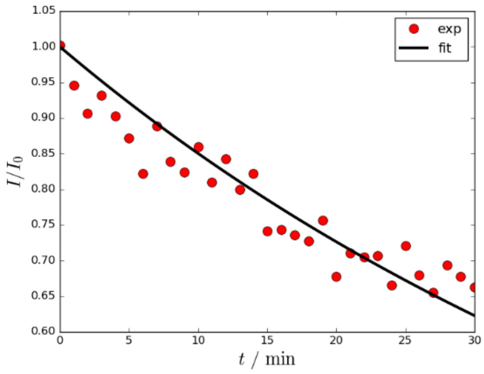

3292	+/- 110 (3.3%)
3252	+/- 133 (4.1%)

ds_DS2_pvp

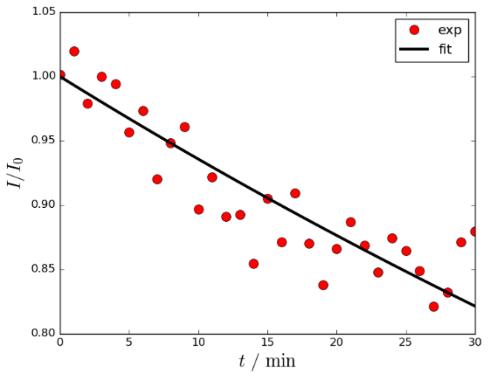

643	+/- 19 (3.0%)
311	+/- 65 (20.9%)

ds_DS3_pvp

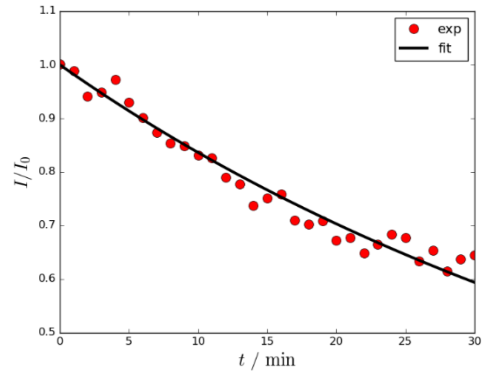

1590	+/- 89 (5.7%)
1425	+/- 69 (4.9%)

6. Fit results in higher salt

Pure PBS_higher salt

ds_DSCtrl_salt

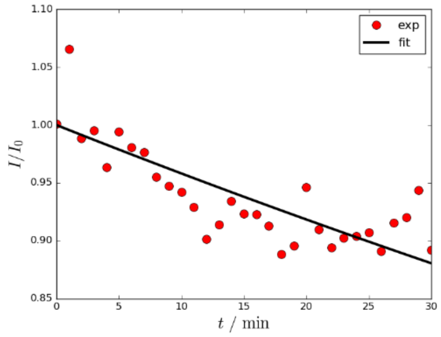

622	+/- 68 (10.9%)
8571	+/- 59 (6.9%)

ds_DS1_salt

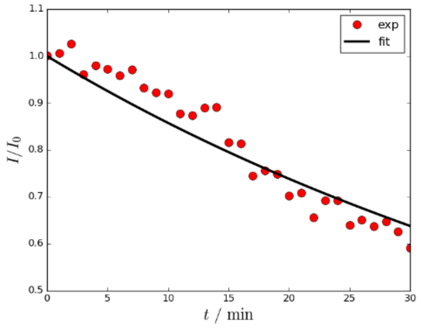

2283	+/- 79 (3.4%)
3117	+/- 127 (4.1%)

ds_DS2_salt

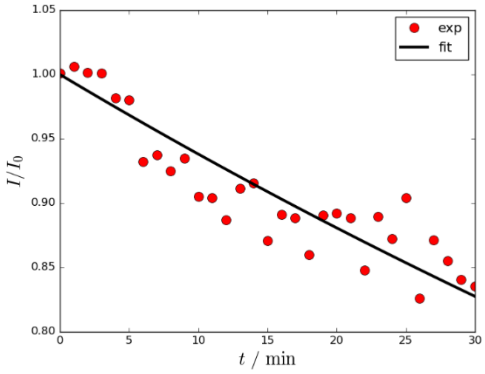

1285	+/- 54.6686840 (4.2%)
1329	+/- 61.9681403 (4.7%)

ds_DS3_salt

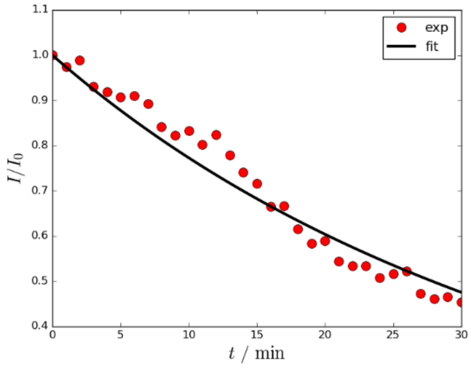

5271	+/- 148 (2.8%)
2157	+/- 174 (8.1%)

Peg buffer_higher salt

ds_DSCtrl_peg_salt

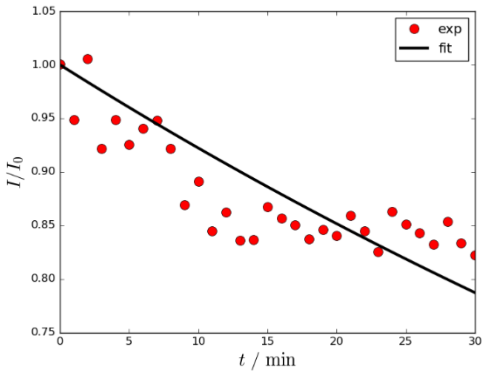

1629	+/- 91 (5.6%)
423	+/- 47 (11.1%)

ds_DS1_peg_salt

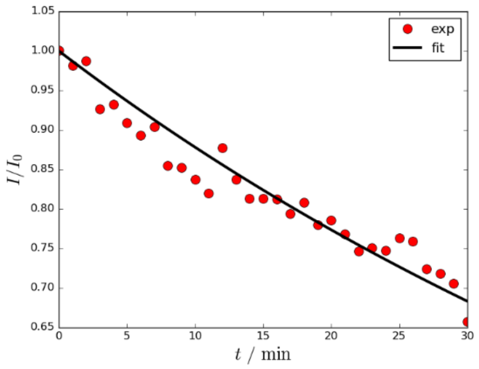

2628	+/- 69 (2.6%)
2352	+/- 129 (5.5%)

ds_DS2_peg_salt

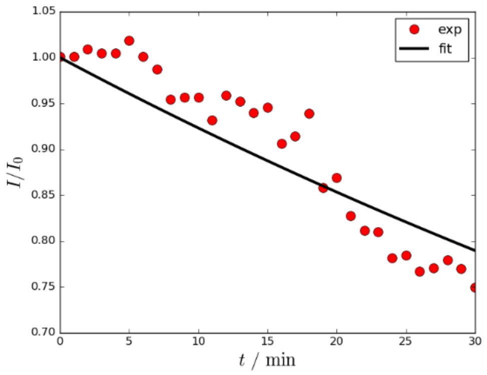

1609	+/- 95 (5.9%)
495	+/- 73 (14.7%)

ds_DS3_peg_salt

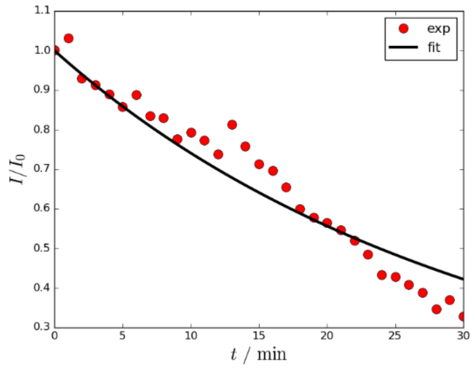

6165	+/- 242 (3.9%)
6558	+/- 368 (5.6%)

Deg buffer_higher salt

ds_DSCtrl_deg_salt

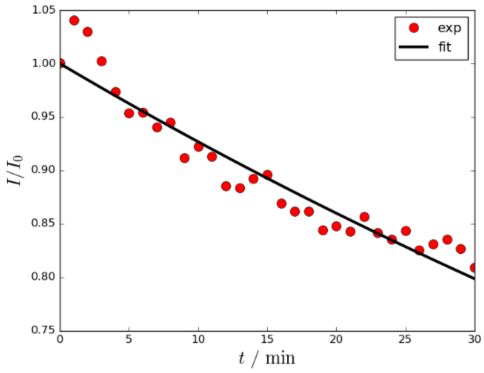

1868	+/- 65 (3.5%)
1529	+/- 46 (3.0%)

ds_DS1_deg_salt

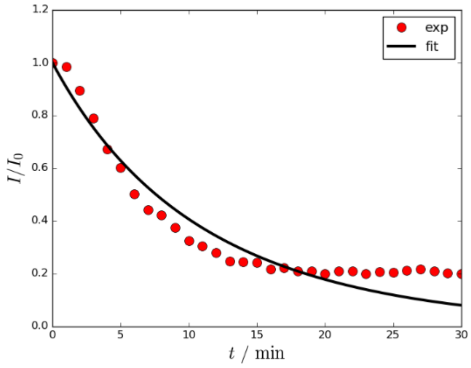

20183	+/- 1101 (5.5%)
19318	+/- 874 (4.5%)

ds_DS2_deg_salt

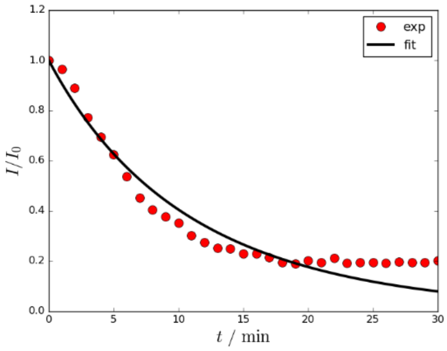

14200	+/- 776 (5.5%)
19409	+/- 785 (4.0%)

ds_DS3_deg_salt

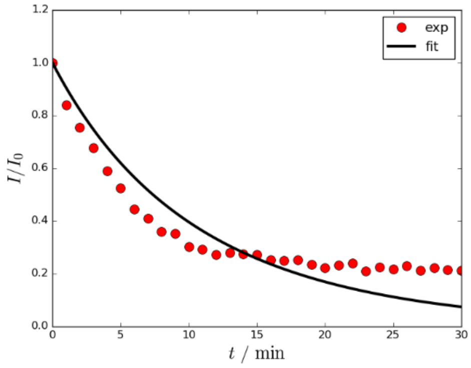

21203	+/- 2064 (9.7%)
19932	+/- 1151 (5.8%)

Peg_deg buffer_higher salt

ds_DSCtrl_peg_deg_salt

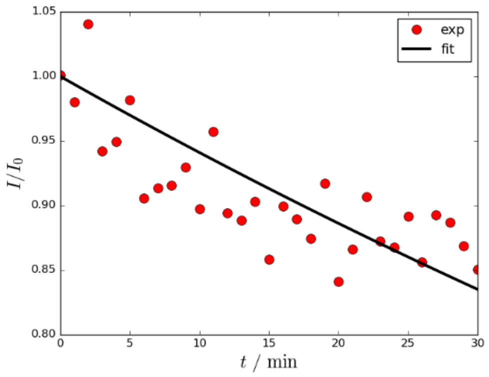

1222	+/- 77 (6.3%)
808	+/- 51 (6.3%)

ds_DS1_peg_deg_salt

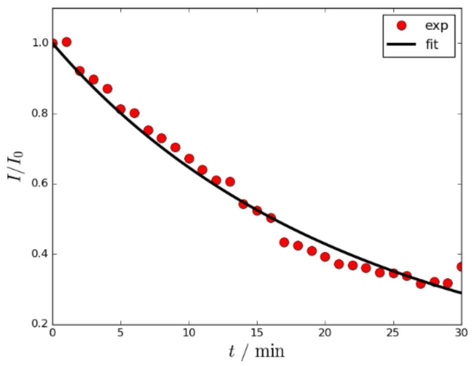

9057	+/- 166 (1.8%)
12243	+/- 388 (3.2%)

ds_DS2_peg_deg_salt

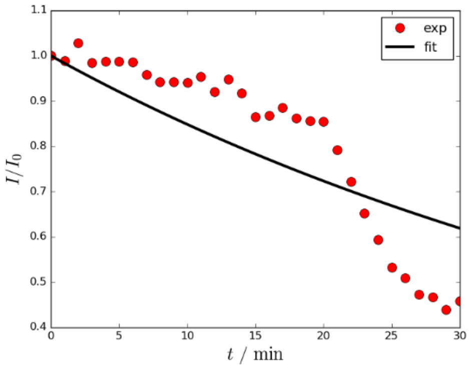

2581	+/- 318 (12.3%)
3335	+/- 341 (10.2%)

ds_DS3_peg_deg_salt

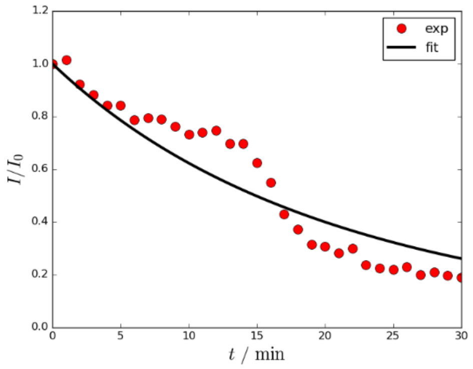

9976	+/- 504 (5.0%)
9855	+/- 592 (6.0%)

Pvp buffer_higher salt

ds_DSCtrl_pvp_salt

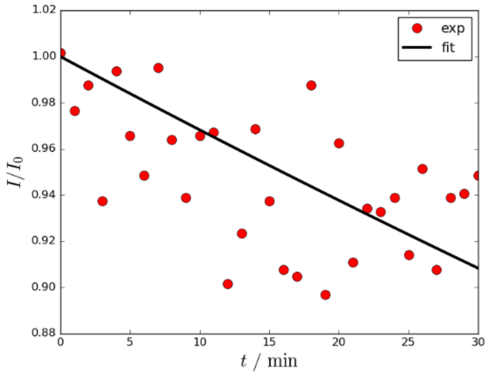

647	+/- 64 (9.9%)
324	+/- 76 (23.5%)

ds_DS1_pvp_salt

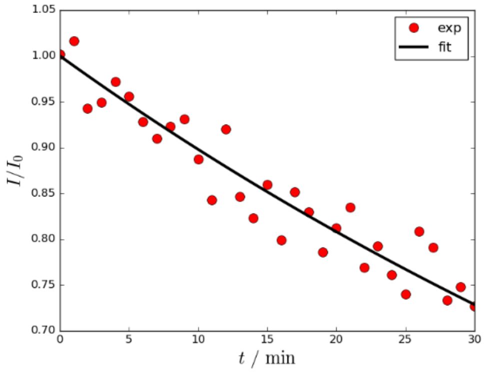

1712	+/- 86 (5.0%)
2172	+/- 69 (3.2%)

ds_DS2_pvp_salt

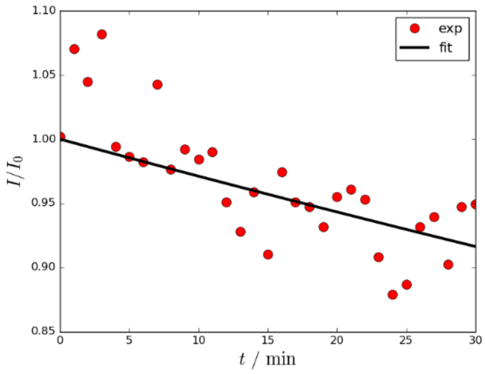

587	+/- 75 (12.8%)
564	+/- 46 (8.2%)

ds_DS3_pvp_salt

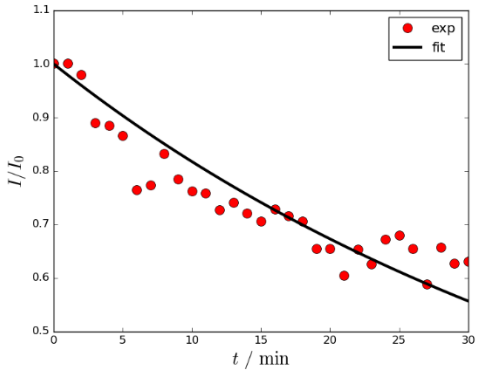

3591	+/- 119 (3.3%)
4103	+/- 169 (4.1%)
